# Thermo-Induced Fluorochromism in Two AIE Zinc Complexes: A Deep Insight into the Structure-Property Relationship

**DOI:** 10.3390/molecules27082551

**Published:** 2022-04-14

**Authors:** Rosita Diana, Ugo Caruso, Francesco Silvio Gentile, Luigi Di Costanzo, Pellegrino Musto, Barbara Panunzi

**Affiliations:** 1 Department of Agriculture, University of Napoli Federico II, Via Università, 100, 80055 Portici, NA, Italy; rosita.diana@unina.it (R.D.); luigi.dicostanzo4@unina.it (L.D.C.); 2 Department of Chemical Sciences, University of Napoli Federico II, Strada Comunale Cinthia, 26, 80126 Napoli, Italy; ugo.caruso@unina.it (U.C.); francesco.gentile@unina.it (F.S.G.); 3 Institute on Polymers Composites and Biomaterials, National Research Council, Via Campi Flegrei, 34, 80078 Pozzuoli, Italy; pellegrino.musto@cnr.it

**Keywords:** fluorochromism, zinc complex, AIEgens, structure-properties relationship

## Abstract

Solid-state emitters exhibiting mechano-fluorochromic or thermo-fluorochromic responses represent the foundation of smart tools for novel technological applications. Among fluorochromic (FC) materials, solid-state emissive coordination complexes offer a variety of fluorescence responses related to the dynamic of noncovalent metal-ligand coordination bonds. Relevant FC behaviour can result from the targeted choice of metal cation and ligands. Herein, we report the synthesis and characterization of two different colour emitters consisting of zinc complexes obtained from *N,O* bidentate ligands with different electron-withdrawing substituents. The two complexes are blue and orange solid-state fluorophores, respectively, highly responsive to thermal and mechanical stress. These emitters show a very different photoluminescent (PL) pattern as recorded before and after the annealing treatment. Through X-ray structural analysis combined with thermal analysis, infrared (IR) spectroscopy, PL, and DFT simulation we provide a comprehensive analysis of the structural feature involved in the fluorochromic response. Notably, we were able to correlate the on-off thermo-fluorochromism of the complexes with the structural rearrangement at the zinc coordination core.

## 1. Introduction

Optical materials with adaptive capabilities to external stimuli (FC active) such as light, pH, temperature, chemical entities, and mechanical stress are critical to the development of novel smart materials. Smart tools are involved in bioengineering and environmental sciences, and in many technological applications, including sensors and actuators [1,2,3,4,5,6,7,8,9,10,11] The molecular emission in optical responsive materials can be trigged by mechanical force or heat. Mechano-fluorochromic (MFC) and thermo-fluorochromic (TFC) materials ensure a variation of the emission colour and/or intensity due to the specific input [12,13,14,15,16,17,18,19,20,21].

Due to their solid-state photoluminescent (PL) properties, many aggregation-induced emission materials (AIEgens) are FC active. MFC AIEgens change their packing structures in presence of external mechanical force [22], undergoing a variation in their PL pattern [1,12,22,23,24,25,26]. In TFC AIEgens the variation of PL behaviour is caused by a thermal treatment inducing a molecular rearrangement [17,27,28]. Both MFC and TFC AIEgens have been used for advanced applications such as display devices, sensing, and optical data storage [17,24,29,30,31,32,33,34,35,36,37,38]. Obviously, the structure–responsiveness relationship is critical for the prediction and understanding of AIEgens’ fluorochromic response. As FC AIEgens can be organic, metallo-organic, or polymeric materials, their response can be ascribed to very different mechanisms [17,38,39,40,41,42,43,44,45]. In the organic AIEgens, the PL change produced upon stimuli relies on the breakage of covalent bonds [46]. On the other hand, AIE active coordination complexes can offer a variety of PL responses due to the dynamic of non-covalent metal-ligand coordination bonds [1,47]. The MFC or TFC behaviour can result from the choice of a suitable metal cation able to vary the coordination environment under mechanical or thermal stress. Specifically, control of the PL properties can be achieved as a function of the coordination architecture and the ligands [11,34,48,49,50,51,52,53,54,55,56,57]. Minimal changes in the ligands can lead to noticeable changes in the chemical and physical properties of coordination complexes, including PL patterns [7,58,59,60,61,62,63,64]. In previous reports [65,66,67,68] we employed zinc (II) cation to activate the luminescence property of *N,O* chelating ligands and achieve a tuneable RGB (red-green-blue) emission by aiming specific substituents. These studies suggested further exploring the structural aspects involved in the PL emission of FC zinc complexes.

**Scheme 1:**
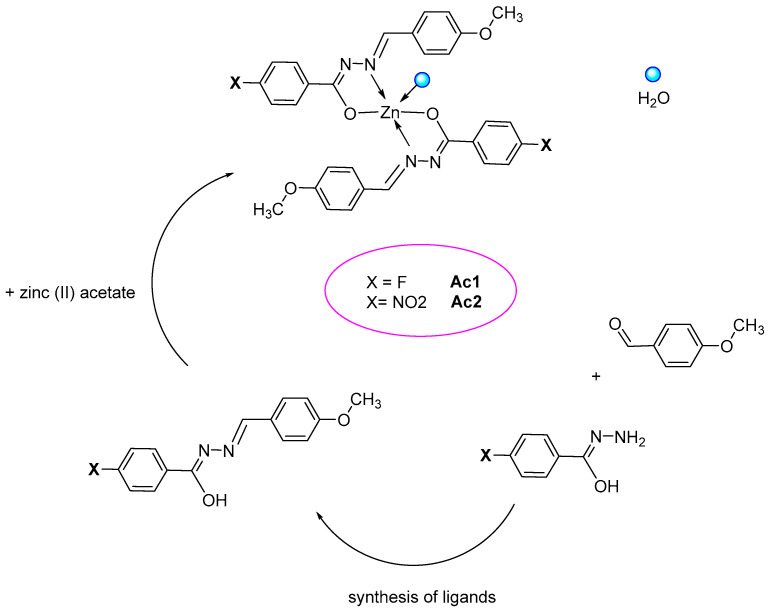
Synthetic route to complexes Ac1 and Ac2.

Herein, we report the synthesis and characterization of two novel zinc complexes obtained from *N,O* bidentate ligands with a different electron-withdrawing substituent. Each substituent was able to selectively direct a certain emission colour. The resulting complexes named Ac1 and Ac2 (acronym of AIE and complex, see Scheme 1) are blue and orange solid-state emitters, respectively. They were found responsive to mechanical stress and highly responsive to thermal stress. As TFC AIEgens, the complexes undergo a decrease in PLQY (PL quantum yield) and a slight red-shift in the PL maximum. Very different emission colours (estimated by naked-eye analysis and CIE diagram) were recorded before and after the annealing treatment. Through X-ray structural analysis, thermal analysis and IR and PL spectroscopy, we provide a comprehensive study of the two complexes’ structural features involved in the TFC response. Density Functional Theory (DFT) formalism was used to analyse and rationalise the experimental data, and to substantiate our findings [69,70]. We could correlate the TFC response with a relevant changeint the zinc coordination sphere. Interestingly, the results underline the complexes’ ability to act in PL on-off mode in a narrow temperature range from two different solid states.

## 2. Results and Discussion

### 2.1. Synthesis and Spectroscopic Analysis

The complexes Ac1 and Ac2 were obtained by reaction of two mononegative ligands with zinc (II) cation. The reaction proceeds with virtually quantitative yields. Single-crystal analysis of the two compounds, NMR, and elemental analysis are in good agreement with the predicted formulas (see Section 2). The ligands are potentially subjected to a keto-enol equilibrium. In presence of zinc (II) (as acetate salt), the more stable keto form [71] turns into the enolate form which acts as a mononegative N,O half-salen pincer [15,71,72,73,74,75]. The poorly emissive organic ligands undergo a fluorescence enhancement according to the CHEF (chelation fluorescence enhancement) effect imposed by zinc (II) coordination [38,76,77]. The optical data recorded by UV-vis and fluorescence spectroscopy in the solid-state are summarised in Table 1 (absorbance and emission maxima in solution are reported in Section 3.1).

**Figure 1:**
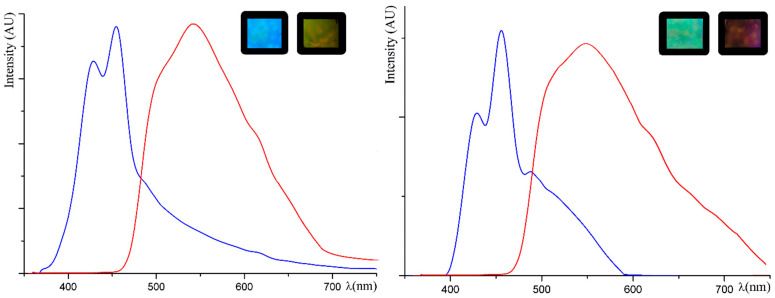
Normalized emission curves were recorded on crystalline thin film samples of Ac1 (blue curve), and Ac2 (red curve) before (on the **left**) and after (on the **right**) the annealing treatment at 140 °C. In the inset the spin-coated thin films used for recording the emission spectra, were photographed under the UV lamp at 365 nm.

In natural light, Ac1 and Ac2 appear as light yellow and yellow powder, respectively. The absorbance pattern of the two crystalline complexes is similar and slightly red-shifted from Ac1 to Ac2 (see maxima in Table 1). On the other hand, the two crystalline complexes are emissive in the solid-state, according to an AIE pattern. Specifically, the cation acts as a constraint locking the poorly emissive organic ligands into a favourable emissive conformation, mostly involving π–π* LCT (ligand charge transfer) transitions [38]. A relevant PL colour tuning based on the different withdrawing substituent (X atom group in Scheme 1) was detected. As expected, [28,68] by increasing the electron-withdrawing strength of X substituent the emission band undergo a relevant red-shift (about 100 nm, see Figure 1) ascribable to a progressive reduction of the gap between the highest occupied molecular orbital (HOMO) and the lowest unoccupied molecular orbital (LUMO) [78].

PLQYs measured on Ac1 and Ac2 crystalline powder are 21% and 5%, respectively. PLQY measured for the nitro derivative is scarce but not negligible, and it runs in agreement with the “energy gap law” effect [75,79], showing a relevant Stokes shift (Table 1). The two crystalline complexes have largely spaced emission CIE coordinates. Naked-eye analysis performed by inspection with a UV lamp reveals Ac1 as a blue emitter and Ac2 as an orange emitter, in the crystalline phase (see inset of Figure 1, left side). 

**Figure 2:**
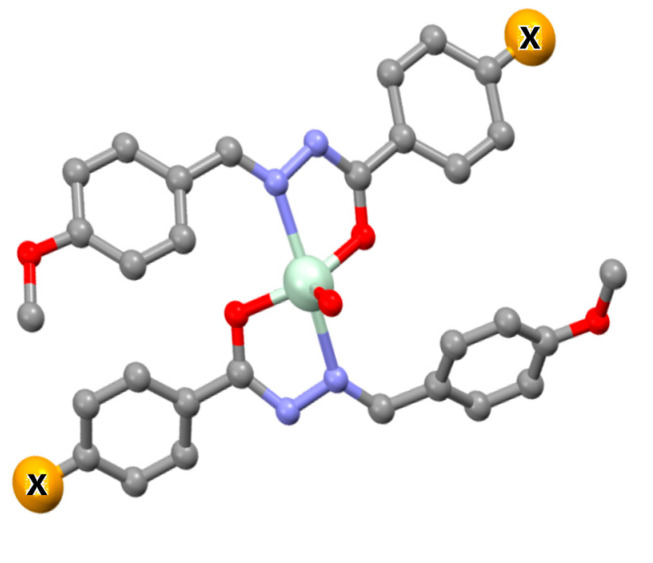
General picture of the crystal structure of Ac1 (X = F) and Ac2 (X = NO_2_).

The compounds were crystallised in various solvents, both anhydrous and not. In solvents such as methanol, diethyl ether, methylene chloride, benzene, and toluene, we obtained irregular and fairly unpure powders to allow further characterization. Contrarily, in many water-soluble solvents like THF, acetone, ethanol, and DMSO, we obtained good quality crystals. Calorimetric and elemental analysis of the regular crystals showed a similar pattern, confirming the presence of a water molecule as observed in crystal structure analysis. We expected that small variations of the crystallisation conditions could drive zinc from tetra- to penta-coordination and vice versa [23,80], but, in our case, the presence of coordinated water molecule in the fifth position seems to yield regular crystals. As confirmed by X-ray diffraction patterns, single crystals with coordinated water were obtained both in THF and in ethanol. Both complexes Ac1 and Ac2 crystallize in a similar monoclinic unit cell with space group C 2/c (as discussed in Section 2.2) with two ligands and a water molecule completing the coordination sphere (Figure 2).

**Figure 3:**
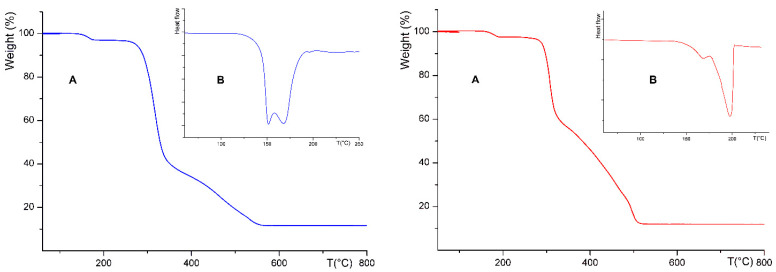
TGA (Section A) and DSC (Section B) curves for Ac1 (blue lines) and Ac2 (red lines) crystalline samples.

The cation displays a trigonal bipyramidal geometry, including one water molecule. The loss of the coordinated water results in the loss of crystallinity of the solid compound and leads to an amorphous phase, established through X-ray diffraction analysis, that in turn is stable for a short temperature range (about 20 °C) and melts afterwards. As the collapsed solid melts, the volatile ligand gets lost. The beginning of the phenomenon is about at the temperature of 140 °C for both complexes, due to their similar structural pattern and involved energy in the water-metal coordination. Starting from about 160 °C both compounds turn into the liquid phase. Optical observations confirmed the persistence of an opaque solid phase for about 20 °C and thermal analyses and X-ray diffraction study were used to ascertain the pattern. In addition, DSC curves registered on both Ac1 and Ac2 evidenced a double-peaked endothermic signal starting from about 140 °C. Based on TGA analysis (see Figure 3) the shoulder could be ascribed to the loss of the coordinated water triggering the collapse of the crystalline architecture and the following state change. For both complexes, TGA curves recorded on the crystalline samples show a weight loss step compatible with the loss of a water molecule in the same temperature range, close to the melting peak and long before the decomposition temperature. X-ray analysis performed at room temperature on thermally treated powders (annealed at 140 °C for 15′ or heated up to 200 °C) of Ac1 and Ac2 shows an amorphous pattern.

Despite the structural and synthetic simpleness, Ac1 and Ac2 provide an intriguing FC behaviour that can be related to the structural properties concerning an ordered and a disordered solid phase. 

**Figure 4:**
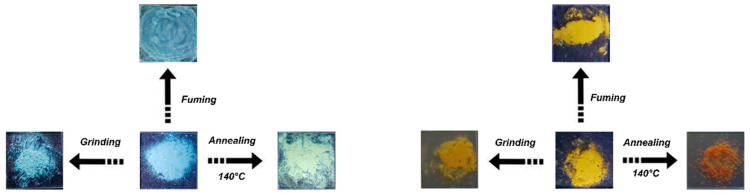
A sample of as-synthetized Ac1 (on the **left**) and Ac2 (on the **right**), photographed after grinding, solvent fuming (acetone) and heating process.

The PL properties of compounds able to form stacked molecular organization are subject to the solid-state molecular packing [81,82,83,84,85]. Therefore, changes in the emission wavelength and/or fluorescence intensity of the materials can be expected. The increase in the crystallite size distribution was expected to produce higher emission intensity due to the hindering of non-radiative relaxation pathways [38]. Both grinding and solvent fuming of the as-synthetized powder are methods to increase the size of particles causing a PL to increase or to disrupt regular crystals achieving the opposite effect [86,87]. In our case, the macroscopic MFC transformations give a less significant optical effect, as seen in Figure 4. We did assume that grinding or fuming processes do not cause significant changes in the molecular arrangement able to justify appreciable PL changes. Contrarily, the thermal treatment (which affects the coordination sphere) does produce a relevant structural modification and a relevant PL modification as consequence. Absorbance and emission pattern, and PLQYs of the samples after grinding and fuming are quite preserved except for the Ac2 sample (after grinding). In this case, the absorbance maximum red-shifts of 6 nm, the emission maximum red-shifts of 10 nm, and PLQY downsize to 4.3%.

On the other hand, a significant TFC response was obtained from Ac1 and Ac2 due to the pivotal role played by the zinc coordination sphere. Ac1 and Ac2 show blue and orange emissions, respectively, in the crystalline phase. As the coordinated water is lost, the crystal collapses and the pentacoordinate core turns gradually into a tetracoordinate fluorogenic unit. It can be assumed that the coordinated water loss has negligible influence on the PL pattern (which is mainly due to the ligands’ electronic transitions). Contrarily, the order degree is relevant to emission behaviour. In the disordered phase, the fluorophores are assumably closer and unstructured. The increased interaction between the fluorophores can cause the PLQY’ to decrease as observed in other studies [88,89,90,91,92] (Table 1). In addition, the molecular interactions specifically affect the PL behaviour of AIEgens. It was demonstrated that intermolecular interactions increasing electronic communication between fluorogenic units can result in the redshift of the emission wavelength [22].

In accordance with the above considerations, by samples annealing at 140 °C and removing the coordinated water molecule (under vacuo of 10-2 Torr for 15′) the emission downsizes to about one third (see Table 1) and undergoes a slight red-shift (see spectra of the treated samples in Figure 1, right side). Specifically, the double-peaked emission spectrum of Ac1 shows the increase of the second band causing a perceived colour more like turquoise, while the emission curve of Ac2 widens towards the NIR area. By extending the thermal treatment up to 200 °C (largely before decomposition, see Section 4), the sample melts. Interestingly, bringing the sample back to room temperature, the process resulted not reversible. The coordinated water is not re-absorbed, and the compounds remain as a frozen glass. The PL pattern recorded on samples treated up to 200 °C is very similar to the samples annealed at 140 °C. Only solvent recrystallization can bring the sample back to the pentacoordinate crystalline form.

Therefore, two structurally and spectroscopically different forms are revealed: the as-obtained samples (pentacoordinated crystalline complexes) and the samples which are obtained starting from 140 °C (amorphous tetracoordinated complexes). The switch between the two states (ordered-disordered) is not reversible and is quite sharp, as confirmed by the IR study. As will be discussed in Section 2.3, FTIR spectra collected on the sample Ac2 change radically just after 140 °C as in a switch mode, confirming the deep transformation of the complex triggered by the coordinated water loss. 

### 2.2. Crystal Structure of Ac1 and Ac2

**Figure 5:**
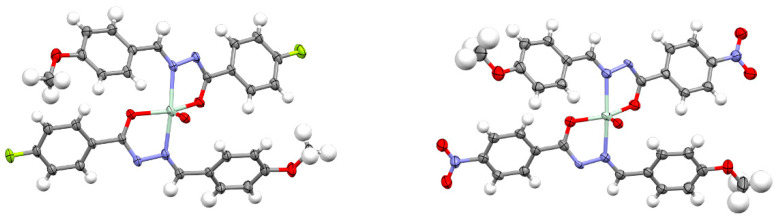
Thermal ellipsoids representation of Ac1 (on the **left**) and Ac2 (on the **right**) drawn at 50% probability Ortep representation. Atoms are color-coded as follows: carbon (brown), hydrogen (white), oxygen (red), nitrogen (blue), zinc (light gray), fluorine (yellow).

Crystals of Ac1 and Ac2 complexes were obtained by slow evaporation at room temperature. The asymmetric unit contains a coordination unit of Ac1 or Ac2 with a water molecule, as shown in Figure 5. Both Ac1 and Ac2 show an overall similar head-to-tail arrangement of two ligand molecules, oriented in opposite directions, and coordinating a single zinc ion. Therefore, zinc recognition is similar between the two complexes despite the different electronic properties of the chemical substituents on the aromatic ring. 

The molecular structure of Ac1 or Ac2 consists of a ligand containing a central acyl hydrazone coordinating segment to zinc ion with an overall *syn*-conformation. The zinc ion lies in the plane of the keto-enol plane of the acyl hydrazone. As a consequence of coordination to zinc ion the X-substituted ring and the methoxybenzene groups are slightly rotated with respect to each other, and with respect to the plane of the acyl hydrazone group. Zinc ion displays a trigonal bipyramidal geometry characterized by two axial nitrogen atom groups (from two ligand molecules) and three oxygen atom groups in the equatorial plane including a water molecule, as shown in Figure 5. Despite the similar overall arrangement, the two complexes show a different orientation of the terminal methoxy groups.

**Figure 6:**
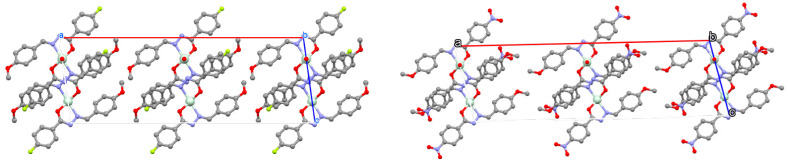
Crystal packing of Ac1 (on the **left**) and Ac2 (on the **right**) showing the assembly of tetramers along the b-axis (water molecule indicated as a red sphere). For sake of clarity, only the Ac2 adduct with higher occupancy is shown. Atoms are color-coded as follows: carbon (brown), hydrogen (white), oxygen (red), nitrogen (blue), zinc (light gray), fluorine (yellow).

The arrangement observed in the asymmetric unit is in turn structured in a tetrameric assembly of two sydimers around a pseudo C2 symmetry. The tetramer is held together by two symmetrically equivalent hydrogen bonds between the equatorial water molecule donating a hydrogen atom group to one of the N- atoms of the hydrazone group. Crystal packing consists of an assembly of these tetramers along the b-axis in agreement with the symmetry elements of the C2/c space group (Figure 6).

### 2.3. FTIR Analysis

FTIR analysis was employed to confirm our hypothesis about the structural transformation involved in the TFC response. We selected Ac2 as the compound with the most relevant TFC behaviour monitoring the IR spectra as a function of temperature and evaluating the absorbance area of the H_2_O band as the temperature rises.

**Figure 7:**
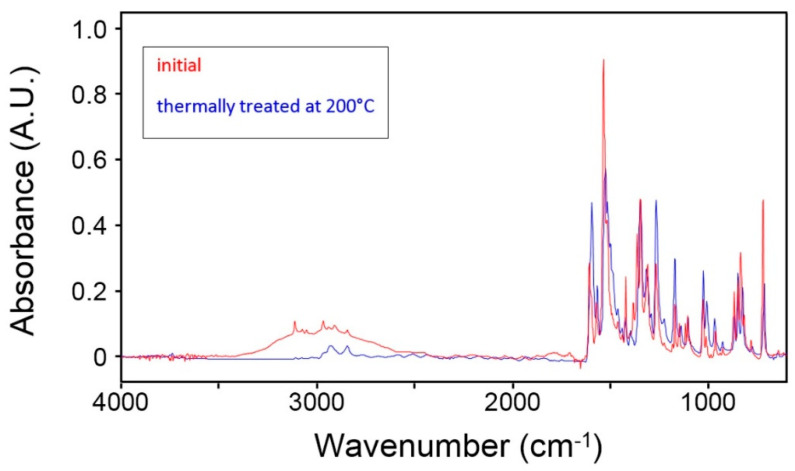
FTIR spectra of the as-prepared sample (red trace) and of the same after exposure to 200 °C under vacuum (10^−2^ Torr). Spectra collected at 30 °C.

**Figure 8:**
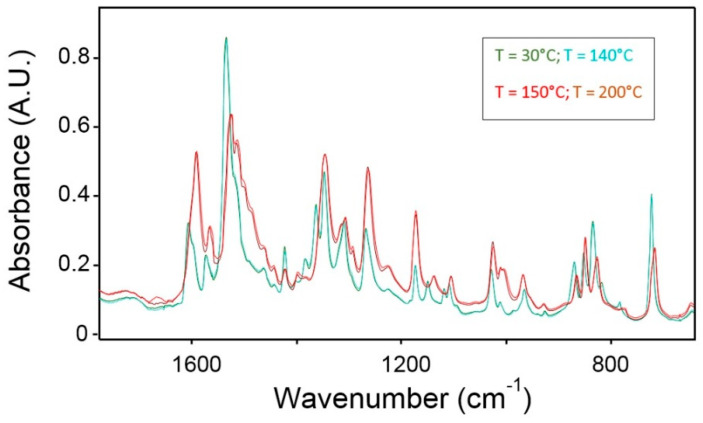
FTIR spectra of Ac2 in the frequency range 1700–600 cm^−1^ collected at 30 °C (green trace), 140 °C (turquoise), 150 °C (red) and 200 °C (light brown).

The FTIR spectrum of the as-prepared Ac2 sample (red trace) and the spectrum of the same complex after thermal treatment at 200 °C under vacuum (blue trace) are reported in Figure 7. Both spectra were collected at room temperature. In the frequency range below 1650 cm^−1,^ the as-prepared sample displays a rich pattern of well-resolved peaks, characteristic of a defect-free, crystalline structure. According to the literature [93] the spectrum is dominated by aromatic modes, i.e., at 1608, 1574 and 1535 cm^−1^ (in-plane ring deformations), at 1172–1118 cm^−1^ [δ(CCH)] and at 965–870 cm^−1^ [*w*(H–Ar)] (note: ν = stretching; δ = bending; *w* = wagging). Two peaks originating from the vibration of the whole tetracoordinated site are identified at 1012 and 834 cm^−1^. The thermal treatment induces a substantial modification of the vibrational pattern. Several peaks disappear (i.e., at 1385, 1150, 1118, 819 cm^−1^); the remaining display conspicuous shift and band broadening. To investigate the thermal behaviour in detail, we performed an in-situ FTIR experiment in which the sample was heated stepwise from 30 to 200 °C, collecting the spectra at each step after thermal stabilization for ten minutes. In Figure 8 are compared the spectra in the frequency range 1700–600 cm^−1^ were collected at 30 °C (green trace), 140 °C (turquoise), 150 °C (red) and 200 °C (light brown). The presence of two different patterns is evident, both well-resolved. 

The spectrum remains well-resolved, and the peak intensities are retained from 150 °C up to 200 °C. These observations suggest an order-disorder transition occurring in the 140–150° interval, which suppresses the peaks characteristic of the crystalline phase (crystal field spitting and/or cooperative reticular modes) and produces a disordered phase which however does not flow up in the sample holder. Interestingly, in the region between 3450 and 2400 cm^−1^, the as-prepared sample displays a broad absorpcentredtered at around 3000 cm^−1^, encompassing a 1000 cm^−1^ interval. Superimposed on this band we found sharp peaks originating from the ν(CH) modes of the aromatics and the methyl group (above and below 3000 cm^−1^, respectively). The band at 3000 cm^−1^ is indicative of the presence of water in the sample: the in-situ FTIR experiment as a function of temperature allowed us to explore in more detail the status of absorbed water.

**Figure 9:**
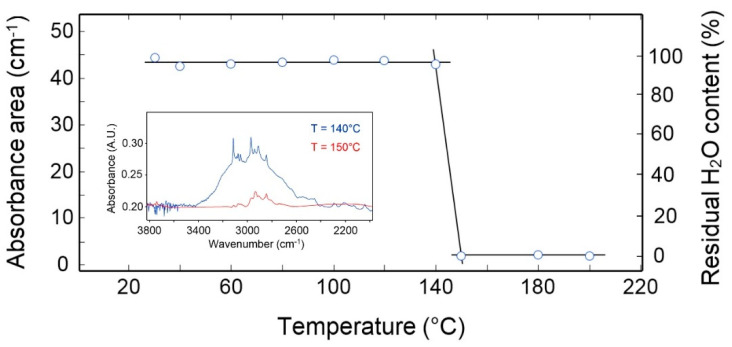
Absorbance area of the H_2_O band at 3000 cm^−1^ and residual water content in the sample as a function of temperature. The inset compares the FTIR spectra in the 3800–2000 cm^−1^ range collected at 140 °C (blue trace) and 150 °C (red trace).

In Figure 9, it is reported that the absorbance area of the H_2_O band, corresponding to the residual water content (WC) in the sample, %WC, as a function of temperature. It is seen that %WC is constant up to 140 °C and suddenly decreases to zero above this temperature (see also an inset of Figure 9). The onset of water desorption corresponds to the transition temperature at which the spectrum in the 1650–600 cm^−1^ interval changes (see Figure 8). These observations suggest that water molecules are incorporated in the crystalline structure of Ac2; they persist if the crystals remain intact and are released as soon as the ordered structure collapses. The order/disorder transition is irreversible: by cooling down the thermally treated sample (10 °C/min), the spectrum collected at 30 °C is coincident (apart from the ordinary temperature effects) to that at 200 °C (see Figure 8). The water band does not re-appear after one week of exposure at atmospheric conditions (25 °C, 40 ± 10 RH), confirming that H_2_O molecules are embedded in the ordered structure during the crystallization process and are not absorbed from the environment.

### 2.4. Theoretical Analysis

**Figure 10:**
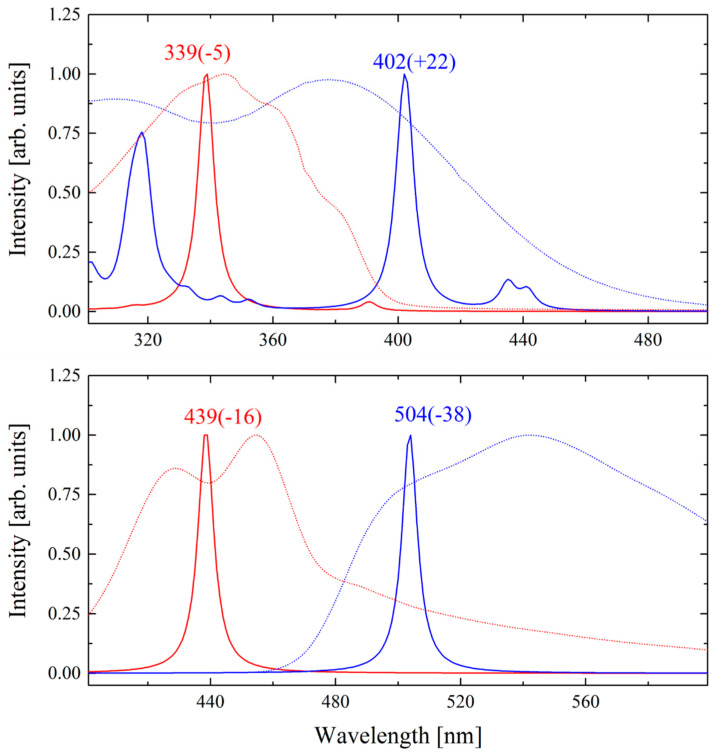
Absorption (**above**) and fluorescence (**below**) spectra of Ac1 (red lines) and Ac2 (blue lines). Calculated data and experimental values are represented by solid and dotted lines, respectively. Simulated excitations were convoluted adopting a Lorentzian function with 6 nm of FWHM. Positions of the main simulated peaks are reported including the difference shift with the experimental values (bracket).

TD-DFT calculations (in Tamm Dancoff approximation) [94] on Ac1 and Ac2 were performed to examine the effect of the electron-withdrawing substituent on the electronic pattern and the PL pattern. The experimental data were compared with the theoretical simulation of both absorbance and emission spectra in the wavelength range of 300–500 nm. Experimental absorption peaks for Ac1 and Ac2 (dotted lines) and the same peaks calculated by TD-DFT simulation (full lines) are reported in Figure 10. The maximum of the band and the trend obtained with the simulation match the experimental trend. For Ac1 the margin of error is minimal (within −1.5% < simulation error < 0.3%) compared to Ac2 (+5.2%). The quality of simulation data is unquestionable, considering: (i) lack of dynamic electronic correlation not included in DFT formalism, (ii) TDDFT treatment not covering orbital relaxation effects [95], (iii) lack of vibronic contribution evaluation and, therefore, the related band broadening effects. Similar considerations are valid for both absorption and emission spectra. The errors in the fluorescence peaks are: −0.8% and +5.2% for Ac1 and Ac2, respectively. Higher systematic errors for Ac2 can be ascribed to a slightly zwitterionic nature of the complex and the charge transfer phenomenon involving the nitro-group [96,97].

The Natural Transition Orbital (NTO) analysis [98] shows the excitations (absorption/emission) involving frontier orbitals HOMO⇒LUMO and HOMO-1⇒LUMO + 1 (see Table 2). The energy position of absorption peaks can be correlated with a decrease of the electronic orbital gap from Ac1 to Ac2 (Table 2), in accordance with the previous discussion (see Section 2.1).

**Figure 11:**
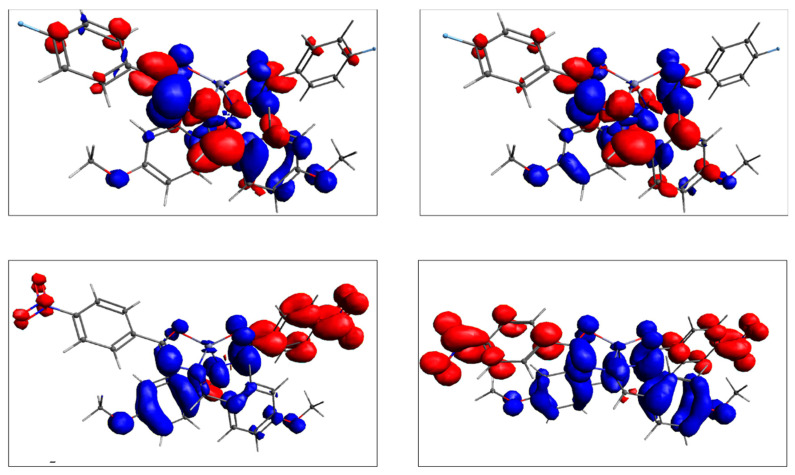
Electron differential density between excited states and ground states in Ac1 (**above**) and Ac2 (**below**). Positive and negative density iso-values are indicated in red and blue, respectively. Differential excitation in the ground state equilibrium geometry (**left**) and excited relaxed geometries (**right**). The iso-surface is calculated at 0.001 |e|/a_0_ for all plots.

The differential densities (ρ^diff^ = ρ^ex^ − ρ^gr^) maps between excited states and ground states (Figure 11) in both cases show a negligible involvement of zinc cation. Although the key role of the metal is as a bridge between two ligands, a negligible superposition of zinc orbital to the overall energy level is observed. Similarly, the fluorine atom in Ac1 shows no contribution to the electron density migration path. Contrarily, the Ac2 system exhibits positive values of isodensities in the substituting sites (Figure 11, red lobes), indicating an electron density transfer from the central region of the molecule to the peripheral zones, both in absorption and in emission phenomena. Interestingly, the absorption phenomenon involves mainly only one of the two ligand molecules in the complex, while the emission phenomenon involves the whole two-ligands system.

## 3. Experimental Section

### 3.1. Synthesis of the Complexes Ac1 and Ac2

The ligands 4-fluoro-*N*′-(4-methoxybenzylidene) benzohydrazide and 4-nitro-*N*′-(4-methoxybenzylidene) benzohydrazide were prepared by reaction of 4-fluorobenzohydrazide or 4-nitrobenzohydrazide, respectively, as previously described [39,40,41,42]. A stoichiometric amount of 4-methoxybenzaldehyde was used. All starting reagents and solvents were commercially purchased from Aldrich products. 

By reacting the related ligand with zinc (II) acetate the two complexes were obtained. As an example, the synthesis of Ac2 is described. An amount of 0.600 g (2.00 mmol) of the ligand 4-nitro-*N*′-(4-methoxybenzylidene) benzohydrazide was dissolved in 12 mL of dry 1,1,2,2, TCE. Zinc (II) acetate (0.183 g, 1.00 mmol) was added to the solution under stirring at 150 °C. After 45 min, the solution was poured into 40 mL of hexane. The precipitated solid was recovered by filtration and purified by crystallization in dichloromethane/hexane. Yield: 70%.

^1^H NMR for Ac1 (400 MHz, DMSO-d6, 25 °C, ppm): 3.81 (s, 3H), 7.00 (d, 2H), 7.21 (t, 2H), 8.03 (dd, 2H), 8.15 (d, 2H), 8.80 (s, 1H). Elemental analysis calculated (%) for C_30_H_26_F_2_N_4_O_5_Zn: C, 57.57; H, 4.19; N, 8.95; found: C, 57.96; H, 4.43; N, 8.69. MALDI-TOF of Ac1 *m*/*z*: 625.18 (M + H). Absorbance and emission NMP solution (excitation wavelength 400 nm): λ_ab_._sol_ = 344 nm; λ_em_._sol_ = 378 nm. Zinc content (calculated ad ZnO% from TGA analysis): experimental = 13.1%; calculated = 12.8%. Mp = 168 °C.

^1^H NMR for Ac2 (400 MHz, DMSO-d6, 25 °C, ppm): 3.81 (s, 3H), 7.05 (d, 2H), 8.17 (d, 2H), 8.19 (d, 2H), 8.27 (d, 2H), 8.87 (s, 1H). ^13^C NMR (400 MHz, DMSO-d6, 25 °C, ppm): 163.2, 158.7, 152.8, 142.5, 141.8, 130.2, 128.3, 126.1, 115.3, 114.6, 55.9, ppm. Elemental analysis calculated (%) for C_30_H_26_N_6_O_9_Zn: C, 52.99; H, 3.85; N, 12.36; found: C, 53.18; H, 3.93; N, 12.51. MALDI-TOF of A1 *m*/*z*: 679.17 (M + H). Absorbance and emission NMP solution (excitation wavelength 400 nm): λ_ab_._sol_ = 455 (429) nm; λ_em_._sol_ = 542 nm. Zinc content (calculated ad ZnO% from TGA analysis): experimental = 12.1%; calculated = 11.8%. Mp = 198 °C.

### 3.2. Materials and Methods

Optical observations were performed employing a Zeiss Axioscop polarizing microscope with an FP90 Mettler heating stage. A DSC scanning calorimeter Perkin Elmer Pyris 1 apparatus at a scanning rate of 10 °C/min under nitrogen flow was used to detect phase transition temperatures and enthalpies. Thermogravimetric analysis was performed by a Perkin Elmer TGA 4000. From TGA analysis the zinc content in models and polymers was measured as ZnO residue. Decomposition temperature was evaluated at 5% weight loss. 1H NMR spectra were recorded in d6 DMSO by using a Bruker Spectrometer 400 MHz operating. Mass spectrometry measurements were performed using a Q-TOF premier instrument (Waters, Milford, MA, USA) equipped with an electrospray ion source and a hybrid quadrupole-time of flight analyzer. Mass spectra were acquired in positive ion mode, in 50% CH_3_CN solution, over the 400–800 *m*/*z* range. Instrument mass calibration was achieved by a separate injection of 1 mM NaI in 50% CH_3_CN. Data were processed by using MassLynx software (Waters). UV-visible absorption spectra and emission fluorescence spectra were recorded by Jasco F-530 spectrometer and by Jasco FP-750 spectrofluorometer, respectively. 

### 3.3. PLQY Calculations

PLQY (the ratio of photons absorbed to photons emitted through fluorescence) of models and polymers were recorded on quartz substrates by a Fluorolog 3 (spectrofluorometer, Horiba Jobin Instruments, Kyoto, Japan). Due to the high refractive index of the films, which results in substantial waveguiding of the luminescence, the spectrofluorometer was equipped with an integrating sphere and an optical fibre connection. This overcomes the angular dependence of the emission from the film. Measurement is done of the fluorescence emission (Ec) and the scatter (Lc) of the sample and the emission and scattering of a blank (La and Ea). From the two spectral measurements (sample and blank), the PLQY can be calculated from the Equation: Φ = Ec − (1 − A)Eb/La·A = Ec − Ea/La − Lc, where Eb is the integrated luminescence from the sample caused by indirect luminescence from the sphere and A is the absorbance of the sample at the excitation wavelength. 

### 3.4. FTIR Apparatus

A stainless steel, vacuum-tight cell equipped with ZnSe windows (Specac HTHP cell) was accommodated in the sample compartment of a suitably modified FTIR spectrometer to perform in-situ acquisition of spectra during heating/cooling cycles. The cell was directly connected through service lines to a water reservoir, a turbo-molecular vacuum pump and a Pirani vacuometer. The cell temperature was electrically controlled in the 35–300 °C range to an accuracy of ±0.5 °C. The FTIR spectrometer was a Spectrum GX from Perkin-Elmer (Norwalk, CT, USA), equipped with a Ge/KBr beam splitter and a wide-band DTGS detector. The transmission spectra were collected with the following instrumental parameters: resolution = 2 cm^−1^; Optical Path Difference (OPD) velocity = 0.5 cm/s; spectral range 4000–650 cm^−1^. The thin film sample used for FTIR analysis was obtained by casting acetone solutions onto KBr tablets in subsequent depositions and dried at room temperature under diaphragm pump vacuo.

### 3.5. Single-Crystal X-ray Analysis

Single crystals of Ac1 and Ac2 complexes were prepared at room temperature by slow evaporation from a solution obtained by mixing stoichiometric amount of the related ligands in ethanol (500 μM, 2 mL) and zinc acetate (II) in water (20 mM, 0.050 mL). Light yellow and yellow coloured diamond-shaped plates of Ac1 and Ac2, respectively, appeared with typical dimensions of 0.07 × 0.1 × 0.6 mm. Small-sized crystals required data collection to be performed from the XRD1 beamline at the Elettra Synchrotron Light Source, Trieste Italy (wavelength, λ = 0.7000 Å). By using a small loop of fine rayon fibre, the selected crystals were dipped in the cryoprotectant paratone oil and flash-frozen in a stream of nitrogen at 100 K. Complete data sets were collected using an oscillation range of 0.5°. Data were processed using XDS and POINTLESS 1.11.21 with a data collection statistic reported in Table 3. Crystals of both complexes gave a similar monoclinic unit cell and space group C 2/c, although, the Ac2 complex shows a slighter bigger unit cell volume (~8%) than the Ac1 complex. No data twinning was detected. Structure solutions of the complexes were found by direct methods using SIR2000 [99] which revealed the presence of one zinc ion in the ASU, located on a centre of symmetry, and most of the expected ligands’ atoms connectivity. Structures were anisotropically refined using full matrix least-squares methods on F2 against all independent measured reflections using SHELXL [100] run under WinGX suite for the refinement of small molecules [101]. A water molecule was found with a fully occupancy coordinated to the zinc ion for both structures. All hydrogen atoms were introduced and refined in agreement with a riding model as implemented in SHELXL. Figures were generated using Mercury CSD 3.6 [102]. During refinement for Ac2 complex a top peak (~7 sigma level) in the residual difference Fourier map was found and near to one of the nitrogen atom groups of the -N-N- bond of the hydrazone planar moiety. This peak could either be interpreted as a disordered nitrogen atom group from the unreacted molecule used for chemical reaction (Scheme 1) or could be the result from X-ray radiation damage used for data collection; however, both interpretations result yield in relatively high values of refinement parameters for Ac2 (Table 3). Similar result was obtained from diffraction of different crystals of Ac2 complex. Crystal data and structure refinement details for the complexes are reported in Table 3 [103]. Crystallographic data for Ac1 and Ac2 and their models were deposited with the Cambridge Crystallographic Data Centre and can be obtained via https://www.ccdc.cam.ac.uk/structures/ (accessed on 14 March 2022).

### 3.6. Molecular Modelling

For each system employed in TD-DFT analysis (adopting ORCA 5.0.1 software) [104] of emission spectra 40 excitation roots have been produced with the corresponding normalized intensities. Basis set and tolerances values are identical to the other adopted in our previous paper [71]. The relative absorption intensities were achieved through the transition electric dipole moments evaluation. A Voigt profile was used to represent the wavelength versus normalized oscillator strength. The data were fit with using a Lorentzian functions, with a Full Width Half Maximum (FWHF) of 30 nm. This convolution yields a broader shape of the absorption spectra and well reproduce the main peaks present in the absorption experimental spectra. The state corresponding to the max intensities of absorption profile were successively relaxed through the gradient evaluation of the specific excited state. Once obtained a converged relaxation of the structures in the excited state, the energies of radiative process S1=>S0 transition (following the Kasha rule) [105] gives an estimation and comparison of the position of emission peaks of the two systems.

## 4. Conclusions

MCF and TFC materials are responsive to severe structural variations and the FC ability is related to metal-ligand dynamic governed by noncovalent coordination bonds. Therefore, the suitable choice of metal cation and ligands can result in a variety of TFC and MCF responses. Herein, we studied two novel zinc complexes easily obtained from half-salen mononegative ligands with a different electron-withdrawing substituent. The simple variation of this substituent produced two complexes with different emission color. In the crystalline form the two complexes are blue and orange solid-state fluorophores, respectively. As for many AIEgens, they resulted responsive to mechanical stress and highly responsive to thermal stress. Specifically, after an annealing treatment the emission curves and the naked-eye perceived colours underwent a red-shift while PLQYs decreased. Through X-ray structural analysis, thermal and IR analysis, PL spectroscopy, and DFT simulation, we were able to understand the relationship between structural details and TFC response of the two complexes. Their FC response was correlated with the structural rearrangement of the zinc coordination core triggered by coordinated water molecule. When this water molecule is lost during annealing process, the regularity of the crystalline structure is transformed in an amorphous phase. In this stage, fluorophores could be closer in space and exposed to radiative relaxation pathways which in turn cause a red-shifted decreased emission. Therefore, we demonstrated as TFC behaviour stems from an irreversible transition order/disorder. Remarkably, the related on-off PL pattern occurring in a small temperature range offers new insights for technological applications as sensing, anti-counterfeiting, and optical thermometry.

## Figures and Tables

**Table 1:** Optical data recorded by UV-vis and fluorescence spectroscopy.

	λ_ab_._film_ (nm) ^[a]^	λ_em_._film_ (nm) ^[b]^	Stokes Shift (nm) ^[c]^	PLQY% ^[d]^	CIE Coord ^[e]^	λ_ab_._film_ (nm) ^[f]^	λ_em_._film_ (nm) ^[g]^	PLQY% ^[h]^	CIE Coord ^[i]^
Ac1	347	429 (455)	92	21 ± 0.2	0.184; 0.154	352	430 (456)	6.8 ± 0.2	0.25; 0.33
Ac2	380	542	162	5.0 ± 0.2	0.421; 0.469	388	549	0.8 ± 0.2	0.57; 0.34

[a] Wavelength of UV-Visible absorbance maxima on spin-coated thin films; [b] Wavelength of emission maxima on spin-coated thin films (excited to absorption maximum wavelength); [c] Stokes shift (from emission to absorption); [d] PLQYs measured on crystalline samples; [e] Emission CIE coordinates of the crystalline samples; [f] Wavelength of UV-Visible absorbance maxima on spin-coated thin films annealed at 140 °C for 15′; [g] Wavelength of emission maxima on spin-coated thin films annealed at 140 °C for 15′ (excited to absorption maximum wavelength); [h] PLQYs measured on the samples annealed at 140 °C for 15′; [i] Emission CIE coordinates of the annealed samples.

**Table 2:** Electronic excitation analysis for Ac1 and Ac2.

	Gap ^[a]^ (eV)	Abs ^[b]^ (nm)	Abs-M.O. ^[c]^	Emi ^[b]^ (nm)	Emi-M.O. ^[c]^
Ac1	3.70	339	H⇒L (0.64); H-1⇒L + 1 (0.33)	439	H⇒L (0.85); H-1⇒L + 1 (0.15)
Ac2	3.21	402	H⇒L (0.94); H-1⇒L + 1 (0.06)	504	H⇒L (0.54); H-1⇒L + 1 (0.45)

[a] Vertical electronic gaps, obtained as the difference from orbitals eigenvalues (Koopmans) are reported in the second column. [b] Abs and Emi are the calculated TD-DFT absorption and emission peaks. The relative orbital compositions in terms of the Natural Transition orbitals are indicated in the adjacent columns. [c] H and L indicates the HOMO and LUMO orbitals and in the round brackets the probability amplitude coefficients.

**Table 3:** Crystal data and structure refinement details for Ac1 and Ac2 complexes.

	Ac1	Ac2
CCDC number	2156626	2156702
Formula complex and solvent	C_30_H_24_N_4_O_4_F_2_·Zn (II)·H_2_O	C_30_H_24_N_6_O_8_ Zn (II)·H_2_O
Temperature (K)	100	100
Wavelength (Å)	0.7000	0.7000
Crystal system	Monoclinic	Monoclinic
Space group	*Χ* 2/c	*Χ* 2/χ
*α* (Å)	30.629 (6)	35.712 (11)
*β* (Å)	8.1810 (16)	7.690 (1)
*χ* (Å)	10.809 (2)	10.954 (1)
β (°)	97.79 (3)	103.703 (12)
R-merge (last shell: 0.75−0.71 Å)	0.046 (0.225)	0.038 (0.169)
CC (1/2)	0.998 (0.967)	0.999 (0.980)
I/σ (I)	12.9 (3.9)	17.4 (5.6)
Completeness (%)	96.7 (95.7)	98.5 (98.8)
Estimated mosaicity (°)	0.21	0.25
Volume	2683.5 (9) Å^3^	2922.6 (10) Å^3^
Z	4	4
Calculated density	1.544 g/cm^3^	1.541 g/cm^3^
*θ* range for data collection (°)	2.35 to 29.754	1.16 to 29.742
Reflections collected/unique	19,218/3996	13,222/4285
Data/restraints/parameters	3996/0/202	4285/0/221
*R1* indices (I > 2σ (I), 3647)	0.0497 (0.0646, all data)	0.1527 (0.1660, all data)
*wR2*	0.153 (0.167, all data)	0.475 (0.484, all data)
Highest diff. peak and hole	1.07; −0.902	5.90; −0.974
*F* (000)	1280	1392
Goodness-of-fit on F^2^	0.93	2.24

## Data Availability

Not applicable.

## Sample Availability

Samples of the compounds are available from authors.

## References

[1] Meng K., Yao C., Ma Q., Xue Z., Du Y., Liu W., Yang D. (2019). A Reversibly Responsive Fluorochromic Hydrogel Based on Lanthanide–Mannose Complex. Adv. Sci..

[2] Zhang F., Zhong H., Chen C., Wu X.-G., Hu X., Huang H., Han J., Zou B., Dong Y. (2015). Brightly Luminescent and Color-Tunable Colloidal CH3NH3PbX3 (X = Br, I, Cl) Quantum Dots: Potential Alternatives for Display Technology. ACS Nano.

[3] Glazer P.J., Leuven J., An H., Lemay S.G., Mendes E. (2013). Multi-stimuli responsive hydrogel cilia. Adv. Funct. Mater..

[4] Diana R., Panunzi B., Tuzi A., Piotto S., Concilio S., Caruso U. (2019). An Amphiphilic Pyridinoyl-hydrazone Probe for Colorimetric and Fluorescence pH Sensing. Molecules.

[5] Diana R., Panunzi B., Shikler R., Nabha S., Caruso U. (2019). A symmetrical azo-based fluorophore and the derived salen multipurpose framework for emissive layers. Inorg. Chem. Commun..

[6] Banerjee D., Hu Z., Li J. (2014). Luminescent metal–organic frameworks as explosive sensors. Dalton Trans..

[7] Bartoli F., Bencini A., Garau A., Giorgi C., Lippolis V., Lunghi A., Totti F., Valtancoli B. (2016). Di- and Triphosphate Recognition and Sensing with Mono- and Dinuclear Fluorescent Zinc(II) Complexes: Clues for the Design of Selective Chemosensors for Anions in Aqueous Media. Chem. Eur. J..

[8] Fan C., Zhang X., Li N., Xu C., Wu R., Zhu B., Zhang G., Bi S., Fan Y. (2020). Zn-MOFs based luminescent sensors for selective and highly sensitive detection of Fe3+ and tetracycline antibiotic. J. Pharm. Biomed. Anal..

[9] Fan L., Wang F., Zhao D., Peng Y., Deng Y., Luo Y., Zhang X. (2019). A self-penetrating and chemically stable zinc (ii)-organic framework as multi-responsive chemo-sensor to detect pesticide and antibiotics in water. J. Mol. Struct..

[10] Gabr M.T., Pigge F.C. (2016). A selective fluorescent sensor for Zn^2+^ based on aggregation-induced emission (AIE) activity and metal chelating ability of bis(2-pyridyl)diphenylethylene. Dalton Trans..

[11] Concilio S., Ferrentino I., Sessa L., Massa A., Iannelli P., Diana R., Panunzi B., Rella A., Piotto S. (2017). A novel fluorescent solvatochromic probe for lipid bilayers. Supramol. Chem..

[12] Prusti B., Samanta P.K., English N.J., Chakravarty M. (2021). A C_3_-symmetric twisted organic salt as an efficient mechano-/thermo-responsive molecule: A reusable and sensitive fluorescent thermometer. Chem. Commun..

[13] Chakraborty M., Chakravarty M. (2021). Variation in solvato-, AIE- And mechano-fluorochromic behavior for furanyl and thiophenyl-substituted anthranyl π-conjugates- And role of tiny flanking donor groups. Mater. Adv..

[14] Suganya S., Debsharma K., Ravindran E., Mahato M.K., Prasad E. (2020). Phenothiazine-Based Mechano-Fluorochromic Gels and Solids: Superhydrophobic Surface Formation and Crystal-to-Crystal Phase Transition. ACS Appl. Polym. Mater..

[15] Wang D., Zhang X., Han X., Zhou Y., Lei Y., Gao W., Liu M., Huang X., Wu H. (2021). Ketone-enol tautomerism, polymorphism, mechanofluorochromism and solid-state acidochromism of isoquinolinone-arylidenehydrazine derivatives. J. Mater. Chem..

[16] Fernández-Mato A., Sánchez-Andújar M., Pato-Doldán B., Señarís-Rodríguez M.A., Platas-Iglesias C., Tordera D., Bolink H.J., Quintela J.M., Peinador C., García M.D. (2014). Spontaneous Self-Assembly of a 1,8-Naphthyridine into Diverse Crystalline 1D Nanostructures: Implications on the Stimuli-Responsive Luminescent Behaviour. Cryst. Growth Des..

[17] Wang L., Chen H., Yin Q., Kang J., Liu B., Weng G., He J. (2020). Fluorochromic polymer films containing ultrasmall silver nanoclusters. Nanotechnology.

[18] Guo P., Liu M., Shi L. (2020). A Zn-based coordination polymer as a luminescent sensor for simple and sensitive detecting of sulfonamides antibiotics and nitroaromatic. J. Solid State Chem..

[19] Casalboni M., Caruso U., De Maria A., Fusco M., Panunzi B., Quatela A., Roviello A., Sarcinelli F., Sirigu A. (2004). New polyurethanes and polyesters for second-order nonlinear optical applications. J. Polym. Sci. Part A: Polym. Chem..

[20] Klongdee F., Youngme S., Boonmak J. (2020). A luminescent sensor based on zinc(II) 1D chain coordination polymer for effective acetone detection. Polyhedron.

[21] Borbone F., Caruso U., Diana R., Panunzi B., Roviello A., Tingoli M., Tuzi A. (2009). Second order nonlinear optical networks with excellent poling stability from a new trifunctional thiophene based chromophore. Organ. Electron..

[22] Yu Y., Xing H., Zhou Z., Liu J., Sung H.H.Y., Williams I.D., Halpert J.E., Zhao Z., Tang B.Z. (2022). How do molecular interactions affect fluorescence behavior of AIEgens in solution and aggregate states?. Sci. China Chem..

[23] Panunzi B., Concilio S., Diana R., Shikler R., Nabha S., Piotto S., Sessa L., Tuzi A., Caruso U. (2018). Photophysical Properties of Luminescent Zinc(II)—Pyridinyloxadiazole Complexes and their Glassy Self-Assembly Networks. Eur. J. Inorg. Chem..

[24] Danilkina N.A., Andrievskaya E.V., Vasileva A.V., Lyapunova A.G., Rumyantsev A.M., Kuzmin A.A., Bessonova E.A., Balova I.A. (2021). 4-Azidocinnoline—Cinnoline-4-amine Pair as a New Fluorogenic and Fluorochromic Environment-Sensitive Probe. Molecules.

[25] Li K., Liu Y., Li Y., Feng Q., Hou H., Tang B.Z. (2017). 2,5-bis(4-alkoxycarbonylphenyl)-1,4-diaryl-1,4-dihydropyrrolo[3,2-b]pyrrole (AAPP) AIEgens: Tunable RIR and TICT characteristics and their multifunctional applications. Chem. Sci..

[26] Roy E., Nagar A., Chaudhary S., Pal S. (2020). Advanced Properties and Applications of AIEgens-Inspired Smart Materials. Ind. Eng. Chem. Res..

[27] Li B., Zhang D., Li Y., Wang X., Gong H., Cui Y.-Z. (2020). A reversible vapor-responsive fluorochromic molecular platform based on coupled AIE–ESIPT mechanisms and its applications in anti-counterfeiting measures. Dye. Pigment..

[28] Butler T., Zhuang M., Fraser C.L. (2018). Color Tuning of Mechanochromic Luminescent β-Diketones via Boron Coordination and Donor-Acceptor Effects. J. Phys. Chem..

[29] Hao H., Ye Z., Dai H., Liu C., Yi A., Xu B., Shi G., Su S., Azad F., Chi Z. (2021). Pyrenyl-Based Aggregation-Induced Emission Luminogen for Highly Sensitive and Selective Detection of 2,4,6-Trinitrotoluene in Water. ChemistrySelect.

[30] Wang R., Diao L., Zhang J., Chen Z., Pu S. (2019). Aggregation-induced emission compounds based on 9,10-dithienylanthracene and their applications in cell imaging. Dye. Pigment..

[31] Panunzi B., Rotiroti L., Tingoli M. (2003). Solvent directed electrophilic iodination and phenylselenenylation of activated alkyl aryl ketones. Tetrahedron Lett..

[32] Hong Y., Lam J.W.Y., Tang B.Z. (2009). Aggregation-induced emission: Phenomenon, mechanism and applications. Chem. Commun..

[33] Kwok R.T.K., Leung C.W.T., Lam J.W.Y., Tang B.Z. (2014). Biosensing by luminogens with aggregation-induced emission characteristics. Chem. Soc. Rev..

[34] Ahmed M., Ibrahim A.-D., Mohamed M.A. (2015). A review on versatile applications of transition metal complexes incorporating Schiff bases. Beni-Suef Univ. J. Basic Appl. Sci..

[35] Alam P., Leung N.L., Zhang J., Kwok R.T., Lam J.W., Tang B.Z. (2020). AIE-based luminescence probes for metal ion detection. Co-ord. Chem. Rev..

[36] Centore R., Panunzi B., Roviello A., Sirigu A., Villano P. (1996). Polymers containing substituted 2-phenyl-benzoxazole side-chain groups: Synthesis and phase behavior. J. Polym. Sci. Part A: Polym. Chem..

[37] Chua M.H., Zhou H., Zhu Q., Tang B.Z., Xu J.W. (2020). Recent advances in cation sensing using aggregation-induced emission. Mater. Chem. Front..

[38] Diana R., Panunzi B. (2021). Zinc (II) and AIEgens: The “Clip Approach” for a Novel Fluorophore Family. A Review. Molecules.

[39] Hirai Y., Laize-Générat L., Wrona-Piotrowicz A., Zakrzewski J., Makal A., Brosseau A., Michely L., Versace D.L., Allain C., Métivier R. (2021). Multi-Directional Mechanofluorochromism of Acetyl Pyrenes and Pyrenyl Ynones. ChemPhysChem.

[40] Liu Y., Liao Y., Ye Z., Chen L., He Y., Huang Y., Lai Y., Chen J., Zhu Q. (2020). Self-reversible mechanofluorochromism of AIE-active C6-unsubstituted tetrahydropyrimidine derivatives. RSC Adv..

[41] Lei S.N., Xiao H., Zeng Y., Tung C.H., Wu L.Z., Cong H. (2020). BowtieArene: A Dual Macrocycle Exhibiting Stimuli-Responsive Fluorescence. Angew. Chem. Int. Ed..

[42] Li Q., Zhu H., Huang F. (2019). Alkyl Chain Length-Selective Vapor-Induced Fluorochromism of Pillar[5]arene-Based Nonporous Adaptive Crystals. J. Am. Chem. Soc..

[43] Wei Y., Wang L., Huang J., Zhao J., Yan Y. (2018). Multifunctional Metallo-Organic Vesicles Displaying Aggregation-Induced Emission: Two-Photon Cell-Imaging, Drug Delivery, and Specific Detection of Zinc Ion. ACS Appl. Nano Mater..

[44] Lescop C. (2017). Coordination-Driven Syntheses of Compact Supramolecular Metallacycles toward Extended Metallo-organic Stacked Supramolecular Assemblies. Acc. Chem. Res..

[45] Qin Y., Peng Q., Chen F., Liu Y., Li K., Zang S. (2021). AIE Ligand Constructed Zn(II) Complex with Reversible Photo-induced Color and Emission Changes. Chem. Res. Chin. Univ..

[46] Xu M., Wang X., Wang Q., Hu Q., Huang K., Lou X., Xia F. (2019). Analyte-responsive fluorescent probes with AIE characteristic based on the change of covalent bond. Sci. China Mater..

[47] Caruso U., Diana R., Panunzi B., Roviello A., Tingoli M., Tuzi A. (2009). Facile synthesis of new Pd(II) and Cu(II) based metallomesogens from ligands containing thiophene rings. Inorg. Chem. Commun..

[48] Terenzi A., Lauria A., Almerico A.M., Barone G. (2014). Zinc complexes as fluorescent chemosensors for nucleic acids: New perspectives for a “boring” element. Dalton Trans..

[49] Chang F.-F., Zhang K., Huang W. (2018). Schiff-base macrocyclic ZnII complexes based upon flexible pendant-armed extended dialdehydes. Dalton Trans..

[50] Tian X., Hussain S., de Pace C., Ruiz-Pérez L., Battaglia G. (2019). Zn II Complexes for Bioimaging and Correlated Applications. Chem. Asian J..

[51] Jayendran M., Begum P.S., Kurup M.P. (2020). Structural, spectral and biological investigations on Cu(II) and Zn(II) complexes derived from NNO donor tridentate Schiff base: Crystal structure of a 1D Cu(II) coordination polymer. J. Mol. Struct..

[52] Wang W.J., Hao L., Chen C.Y., Qiu Q.M., Wang K., Song J.B., Li H. (2017). Red-shift in fluorescence emission of D-A type asymmetrical Zn(II) complexes by extending the π-π stacking interaction. RSC Adv..

[53] Xie Y.-Z., Shan G.-G., Li P., Zhou Z.-Y., Su Z.-M. (2013). A novel class of Zn(II) Schiff base complexes with aggregation-induced emission enhancement (AIEE) properties: Synthesis, characterization and photophysical/electrochemical properties. Dye. Pigment..

[54] Feng G., Zhang C.-J., Lu X., Liu B. (2017). Zinc(II)-Tetradentate-Coordinated Probe with Aggregation-Induced Emission Characteristics for Selective Imaging and Photoinactivation of Bacteria. ACS Omega.

[55] Kursunlu A.N., Ozmen M., Güler E. (2019). A Novel Fluorescent Chemosensor for cu (II) Ion: Click Synthesis of Dual-Bodipy Including the Triazole Groups and Bioimaging of Yeast Cells. J. Fluoresc..

[56] Uysal S., Kursunlu A.N. (2011). The Synthesis and Characterization of Star Shaped Metal Complexes of Triazine Cored Schiff Bases: Their Thermal Decompositions and Magnetic Moment Values. J. Inorg. Organomet. Polym. Mater..

[57] Borbone F., Caruso U., Causà M., Fusco S., Panunzi B., Roviello A., Shikler R., Tuzi A. (2014). Series of O,N,O-tridentate ligands zinc(II) complexes with high solid-state photoluminescence quantum yield. Eur. J. Inorg. Chem..

[58] Zhang W., Zhong X. (2011). Facile synthesis of ZnS-CuInS2-alloyed nanocrystals for a color-tunable fluorchrome and photocatalyst. Inorg. Chem..

[59] Li S., Wen H., Yuan N., Xie P., Qin J., Wang Z. (2020). Synthesis, characterization and computational studies of Zn complex based on the 8-hydroxyquinoline group containing benzimidazole. RSC Adv..

[60] Miguez F., Menzonatto T.G., Netto J.F.Z., Souza-Silva I.M., Verano-Braga T., Lopes J.F., De Sousa F.B. (2020). Photo-dynamic and fluorescent zinc complex based on spiropyran ligand. J. Mol. Struct..

[61] Diana R., Panunzi B. (2020). The Role of Zinc(II) Ion in Fluorescence Tuning of Tridentate Pincers: A Review. Molecules.

[62] Xu B., Chi Z., Zhang X., Li H., Chen C., Liu S., Zhang Y., Xu J. (2011). A new ligand and its complex with multi-stimuli-responsive and aggregation-induced emission effects. Chem. Commun..

[63] Fan C., Wang L., Xu C., Wu R., Li N., Zhang D., Zhang X., Bi S., Fan Y. (2020). Synthesis, structure diversity, and dye adsorption and luminescent sensing properties of Zinc (II) coordination polymers based on 1,3,5-tris(1-imidazolyl)benzene and 1,3-bis(1-imidazolyl)toluene. J. Solid State Chem..

[64] Kursunlu A.N. (2015). Synthesis and photophysical properties of modifiable single, dual, and triple-boron dipyrromethene (Bodipy) complexes. Tetrahedron Lett..

[65] Panunzi B., Diana R., Caruso U. (2019). A Highly Efficient White Luminescent Zinc (II) Based Metallopolymer by RGB Approach. Polymers.

[66] Diana R., Panunzi B., Concilio S., Marrafino F., Shikler R., Caruso T., Caruso U. (2019). The effect of bulky substituents on two π-conjugated mesogenic fluorophores. Their organic polymers and zinc-bridged luminescent networks. Polymers.

[67] Diana R., Panunzi B., Shikler R., Nabha S., Caruso U. (2019). Highly efficient dicyano-phenylenevinylene fluorophore as polymer dopant or zinc-driven self-assembling building block. Inorg. Chem. Commun..

[68] Borbone F., Caruso U., Di Palma S., Fusco S., Nabha S., Panunzi B., Shikler R. (2015). High Solid State Photoluminescence Quantum Yields and Effective Color Tuning in Polyvinylpyridine Based Zinc(II) Metallopolymers. Macromol. Chem. Phys..

[69] Panunzi B., Borbone F., Capobianco A., Concilio S., Diana R., Peluso A., Piotto S., Tuzi A., Velardo A., Caruso U. (2017). Synthesis, spectroscopic properties and DFT calculations of a novel multipolar azo dye and its zinc(II) complex. Inorg. Chem. Commun..

[70] Borbone F., Caruso U., Concilio S., Nabha S., Piotto S., Shikler R., Tuzi A., Panunzi B. (2017). From cadmium(II)-aroylhydrazone complexes to metallopolymers with enhanced photoluminescence. A structural and DFT study. Inorg. Chim. Acta.

[71] Diana R.C., Gentile U., Di Costanzo F.S., Musto L., Barbara Panunzi P. (2022). Structural feature of thermo-induced fluorochromism in a 1D zinc coordination polymer. A cross-analysis by PL and FTIR spectroscopy, and DFT formalism. Dyes Pigm..

[72] Annaraj B., Pan S., Neelakantan M., Chattaraj P.K. (2014). DFT study on the ground state and excited state intramolecular proton transfer of propargyl arm containing Schiff bases in solution and gas phases. Comput. Theor. Chem..

[73] Panunzi B., Diana R., Concilio S., Sessa L., Tuzi A., Piotto S., Caruso U. (2019). Fluorescence pH-dependent sensing of Zn(II)by a tripodal ligand. A comparative X-ray and DFT study. J. Luminesc..

[74] Fita P., Luzina E., Dziembowska T., Kopeć D., Piątkowski P., Radzewicz C., Grabowska A. (2005). Keto–enol tautomerism of two structurally related Schiff bases: Direct and indirect way of creation of the excited keto tautomer. Chem. Phys. Lett..

[75] Panunzi B., Diana R., Concilio S., Sessa L., Shikler R., Nabha S., Tuzi A., Caruso U., Piotto S. (2018). Solid-State Highly Efficient DR Mono and Poly-dicyano-phenylenevinylene Fluorophores. Molecules.

[76] Patra L., Das S., Gharami S., Aich K., Mondal T.K. (2018). A new multi-analyte fluorogenic sensor for efficient detection of Al^3+^ and Zn^2+^ ions based on ESIPT and CHEF features. New J. Chem..

[77] Mei J., Leung N.L.C., Kwok R.T.K., Lam J.W.Y., Tang B.Z. (2015). Aggregation-Induced Emission: Together We Shine, United We Soar!. Chem. Rev..

[78] Argeri M., Borbone F., Caruso U., Causà M., Fusco S., Panunzi B., Roviello A., Shikler R., Tuzi A. (2014). Color tuning and noteworthy photoluminescence quantum yields in crystalline mono-/dinuclear ZnII complexes. Eur. J. Inorg. Chem..

[79] Caruso U., Panunzi B., Diana R., Concilio S., Sessa L., Shikler R., Nabha S., Tuzi A., Piotto S. (2018). AIE/ACQ Effects in Two DR/NIR Emitters: A Structural and DFT Comparative Analysis. Molecules.

[80] Li Y., Yang Z., Song B., Xia H., Wang Z. (2017). Syntheses, crystal structures, and fluorescent studies of Cu(II) and Zn(II) complexes bearing 2-acetonaphthonebenzoylhydrazone ligand. Inorg. Nano-Metal Chem..

[81] Salimimarand M., La D., Al Kobaisi M., Bhosale S.V. (2017). Flower-like superstructures of AIE-active tetraphenylethylene through solvophobic controlled self-assembly. Sci. Rep..

[82] Salimimarand M., La D.D., Bhosale S.V., Jones L.A., Bhosale S.V. (2017). Influence of Odd and Even Alkyl Chains on Supramolecular Nanoarchitecture via Self-Assembly of Tetraphenylethylene-Based AIEgens. Appl. Sci..

[83] Caruso U., Panunzi B., Roviello G.N., Roviello G., Tingoli M., Tuzi A. (2009). Synthesis, structure and reactivity of amino-benzodifurane derivatives. C. R. Chim..

[84] Zheng H.-W., Wu M., Yang D.-D., Liang Q.-F., Li J.-B., Zheng X.-J. (2021). Multistimuli Responsive Solid-State Emission of a Zinc(II) Complex with Multicolour Switching. Inorg. Chem..

[85] Wang L., Zhang R., Huang Z., Guo S., Yang J.-X., Kong L. (2021). A multi-stimuli-responsive tetraphenylethene derivative with high fluorescent emission in solid state. Dye. Pigment..

[86] Chen Z., Liu G., Wang R., Pu S. (2017). Highly emissive carbazole-based gold(i) complex with a long room-temperature phosphorescence lifetime and self-reversible mechanochromism characteristics. RSC Adv..

[87] Yang Y., Yang X.G., Fang X., Wang K.-Z., Yan D. (2018). Reversible Mechanochromic Delayed Fluorescence in 2D Metal-Organic Micro/Nanosheets: Switching Singlet-Triplet States through Transformation between Exciplex and Excimer. Adv. Sci..

[88] Ren F., Shi J., Tong B., Cai Z., Dong Y. (2019). Effects of fused rings linked to the 2,5-position of pyrrole derivatives with near-infrared emission on their aggregation-enhanced emission properties. Mater. Chem. Front..

[89] Caruso U., Panunzi B., Roviello A., Tingoli M., Tuzi A. (2011). Two aminobenzothiazole derivatives for Pd(II) and Zn(II) coordination: Synthesis, characterization and solid state fluorescence. Inorg. Chem. Commun..

[90] Roviello A., Borbone F., Carella A., Diana R., Roviello G., Panunzi B., Ambrosio A., Maddalena P. (2009). High quantum yield photoluminescence of new polyamides containing oligo-PPV amino derivatives and related oligomers. J. Polym. Sci. Part A Polym. Chem..

[91] Wei F., Fang L., Huang Y. (2010). Synthesis, characterization, crystal structures, and photophysical properties of a series of room-temperature phosphorescent copper(I) complexes with oxadiazole-derived diimine ligand. Inorg. Chim. Acta.

[92] Caruso U., Panunzi B., Roviello A., Tuzi A. (2013). Fluorescent metallopolymers with Zn(II) in a Schiff base/phenoxide coordination environment. Inorg. Chem. Commun..

[93] Singh P., Singh D.P., Tiwari K., Mishra M., Singh A.K., Singh V.P. (2015). Synthesis, structural investigations and corrosion inhibition studies on Mn(ii), Co(ii), Ni(ii), Cu(ii) and Zn(ii) complexes with 2-amino-benzoic acid (phenyl-pyridin-2-yl-methylene)-hydrazide. RSC Adv..

[94] Hirata S., Head-Gordon M. (1999). Time-dependent density functional theory within the Tamm–Dancoff approximation. Chem. Phys. Lett..

[95] Ronca E., Angeli C., Belpassi L., De Angelis F., Tarantelli F., Pastore M. (2014). Density Relaxation in Time-Dependent Density Functional Theory: Combining Relaxed Density Natural Orbitals and Multireference Perturbation Theories for an Improved Description of Excited States. J. Chem. Theory Comput..

[96] Dreuw A.A., Head-Gordon M. (2004). Failure of Time-Dependent Density Functional Theory for Long-Range Charge-Transfer Excited States: The Zincbacteriochlorin−Bacteriochlorin and Bacteriochlorophyll−Spheroidene Complexes. J. Am. Chem. Soc..

[97] Hamel S., Duffy P., Casida M.E., Salahub D.R. (2002). Kohn–Sham orbitals and orbital energies: Fictitious constructs but good approximations all the same. J. Electron Spectrosc. Relat. Phenom..

[98] Wu Q., Yang W. (2003). A direct optimization method for calculating density functionals and exchange–correlation potentials from electron densities. J. Chem. Phys..

[99] Burla M.C., Carrozzini B., Cascarano G.L., Giacovazzo C., Polidori G. (2017). Solving proteins at non-atomic resolution by direct methods: Update. J. Appl. Crystallogr..

[100] Sheldrick G.M. (2015). SHELXT-Integrated space-group and crystal-structure determination. Acta Crystallogr. Sec. Found. Crystallogr..

[101] Farrugia L.J. (2012). WinGX and ORTEP for Windows: An update. J. Appl. Crystallogr..

[102] MacRae C.F., Sovago I., Cottrell S.J., Galek P.T.A., McCabe P., Pidcock E., Platings M., Shields G.P., Stevens J.S., Towler M. (2020). Mercury 4.0: From visualization to analysis, design and prediction. J. Appl. Crystallogr..

[103] Evans P.R. (2011). An introduction to data reduction: Space-group determination, scaling and intensity statistics. Acta Crystallogr. Sec. D Biol. Crystallogr..

[104] Neese F. (2018). Software update: The ORCA program system, version 4.0. Wiley Interdiscip. Rev. Comput. Mol. Sci..

[105] Kasha M. (1950). Characterization of electronic transitions in complex molecules. Discuss. Faraday Soc..

